# Evaluation of Lactic Acid Bacteria on the Inhibition of *Vibrio parahaemolyticus* Infection and Its Application to Food Systems

**DOI:** 10.3390/molecules23051238

**Published:** 2018-05-22

**Authors:** Cheng-Chih Tsai, Yung-Hsien Hung, Lan-Chun Chou

**Affiliations:** Department of Food Science and Technology, HungKuang University, No. 1018, Sec. 6, Taiwan Boulevard, Shalu District, Taichung City 43302, Taiwan; norst.tsai@gmail.com (Y.-H.H.); yles6428@yahoo.com.tw (L.-C.C.)

**Keywords:** lactic acid bacteria, *Vibrio parahaemolyticus*, cytokines, inflammation, adhesion

## Abstract

This study tested the effect of lactic acid bacteria (LAB) inhibition on *Vibrio parahaemolyticus* BCRC (Bioresource Collection and Research Center) 10806 and BCRC 12865 in a food model. MTT [3-(4,5-Dimethylthiazol-2-yl)-2,5-diphenyltetrazolium bromide] assays indicated that Caco-2 cells were not damaged after a two-hour treatment with lactic acid bacteria (LAB) and *V. parahaemolyticus*. The LAB cell culture and supernatant effectively inhibited the growth of *V. parahaemolyticus* in a food model. ELISA (Enzyme-linked immunosorbent assay) results indicated the significant inhibition of TNF-α; IL-1β; and IL-6; but *Lactobacillus plantarum* PM 222 and *L. plantarum* LP 735 did not significantly affect IL-8 levels. Real-time polymerase chain reaction (PCR) results indicated that LAB could inhibit the mRNA expression of proinflammatory cytokines IL-8; IL-6; and TNF-α; which were induced by *V. parahaemolyticus*. After rat-received LAB; the expression levels of TNF-α; IL-6; and IL-8 in the serum decreased significantly. In intestinal histology; the rat that received *L. plantarum* PM 222 and *L. plantarum* LP 010 was able to alleviate the intestinal villi damage caused by *V. parahaemolyticus*; which also helped reduce cell apoptosis. In conclusion; our results indicate that LAB can inhibit inflammatory responses caused by *V. parahaemolyticus* and can effectively inhibit the growth of *V. parahaemolyticus* in food products.

## 1. Introduction

*Vibrio parahaemolyticus* is a Gram-negative, halophilic, and facultative anaerobic bacterium that is widely distributed in brackish saltwater. *V. parahaemolyticus* causes foodborne gastroenteritis in all coastal countries and is the most common foodborne pathogen in Taiwan. Infection is mainly caused by the ingestion of seafood, and an infection with more than 10^5^ colony forming units (CFU)/g of bacteria causes pathogenicity. The clinical signs of *V. parahaemolyticus* gastroenteritis include diarrhea, abdominal pain, nausea, vomiting, headache, fever, and shivering [1]. Patients with severe clinical symptoms of *V. parahaemolyticus* are treated with antibiotics such as chloramphenicol, cephalosporin, or tetracycline.

Shirazinejad et al. evaluated the effect of lactic acid application on fresh shrimp against *V. parahaemolyticus*. The results showed that *V. parahaemolyticus* was reduced by more than two log CFU/g after a 10 min immersion in 3% (*v*/*v*) lactic acid in shrimp, and no adverse changes in the sensory characteristics of the shrimp were observed [2]. Terzi et al. studied artificially contaminated mussels after 15 min immersion in 1% (*v*/*v*) lactic acid, with a reduction in the viability of *V. parahaemolyticus* greater than 3.38 log CFU/g [3]. Hwanhlem et al. stated that probiotic lactic acid bacteria (LAB) can completely inhibit the growth of *V. parahaemolyticus* within 24 h [4]. Xi added *Lactobacillus plantarum* ATCC (American Type Culture Collection) 8014 to artificial seawater to purify Pacific oysters and found that *V. parahaemolyticus* in oysters was significantly reduced (over 3.42 MPN/g) after five days of purging at 10 ± 1 °C, indicating lactobacilli can be applied to seafood purification at low temperatures to reduce *V. parahaemolyticus* [5].

Liu et al. demonstrated that *Bacillus subtilis* E20 (10^8^ CFU/g feed) as a feed additive can effectively reduce the mortality of groupers infected with *Iridovirus* [6]. Cha et al. tested the survival of flounder (*Paralichthys olivaceus*) exposed to the pathogen *Stretococus iniae* and found that the diets with supplementation of *B. subtilis* resulted in lower mortality rates [7]. Probiotics typically affect the production of cytokines and chemokines, such as TNF-α, IL-6, IL-10, IL-12, and IFN-c, by innate and acquired immunity [8,9]. He et al. determined that *B. subtilis* C-3102 could enhance the adhesion of intestinal bacteria to the intestinal mucosa surface of *Oreochromis niloticus* ♀ × *O. aureus* ♂ and also enhance the secretion of intestinal cytokines such as IL-1β, TGF-β, and TNF-α [10].

In this study, we investigated whether LAB could inhibit the growth of *V. parahaemolyticus* in aquatic products using the sea bream fillet as a carrier of seafood products. ELISA and reverse transcription polymerase chain reaction (RT-PCR) were used to determine the concentration of inflammatory markers and mRNA expression in Caco-2, Raw 264.7, and HT-29 cells. Finally, BALB/c mice were used to explore the mechanism of LAB inhibition of intestinal inflammation caused by *V. parahaemolyticus*. Hematoxylin and eosin stain, and Terminal deoxynucleotidyl transferase dUTP nick end labeling (TUNEL) stain, were used to observe the intestinal cell damage and apoptosis caused by inflammation.

## 2. Results

### 2.1. Survival Rate of Caco-2 Cells Co-Cultured with V. parahaemolyticus and LAB

**Figure 1:**
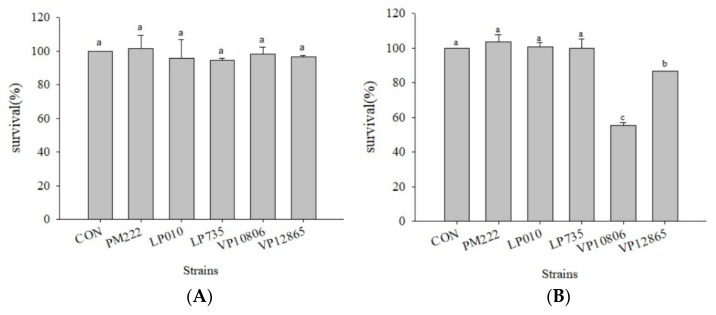
The survival rates of caco-2 cell line co-cultured with the *V. parahaemolyticus* and lactic acid bacteria strains for (**A**) two hours and (**B**) four hours. ^a,b,c^ values in the same column with different superscripts mean significant difference (*p* < 0.05) using the Duncan’s multiple range test.

In this experiment, MTT [3-(4,5-Dimethylthiazol-2-yl)-2,5-diphenyltetrazolium bromide] assays were used to analyze the effect of LAB and *V. parahaemolyticus* on Caco-2 viability. The results showed that LAB and *V. parahaemolyticus* did not significantly damage cells after two hours of co-culture (Figure 1A). Four hours after co-cultivation, *V. parahaemolyticus* BCRC 10806 and BCRC 12865 harmed the cells, with BCRC 10806 causing the most significant damage. There was no difference in cell viability between the three strains of LAB (*L. plantarum* PM 222, LP 010, and LP 735) (Figure 1B).

### 2.2. LAB Suppresses V. parahaemolyticus in Food Mode

The growth of *V. parahaemolyticus* BCRC 10806 and BCRC 12865 were inhibited by co-culturing with LAB cells or supernatant of three LAB strains (*L. plantarum* PM 222, LP 010, and LP 735) at 4 °C for four hours (Table 1). Two strains of *V. parahaemolyticus* were also significantly inhibited after being co-cultured with the three strains (*L. plantarum* PM 222, LP 010, and LP 735) of LAB cells or their supernatant at room temperature for four hours (Table 1). Three LAB strains survived optimally after four hours of co-cultivation at 4 °C and room temperature (Table 2).

### 2.3. LAB Inhibit V. parahaemolyticus-Induced Secretion of Inflammatory Cytokines by HT-29 Intestinal Epithelial Cells or RAW 264.7 Macrophages

**Figure 2:**
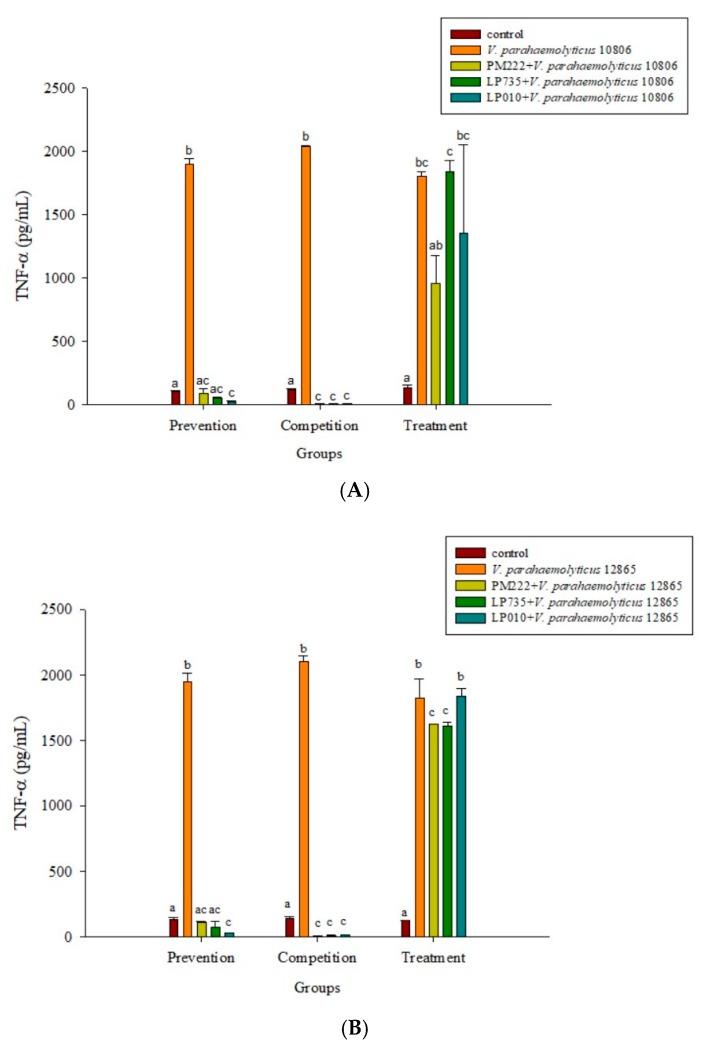

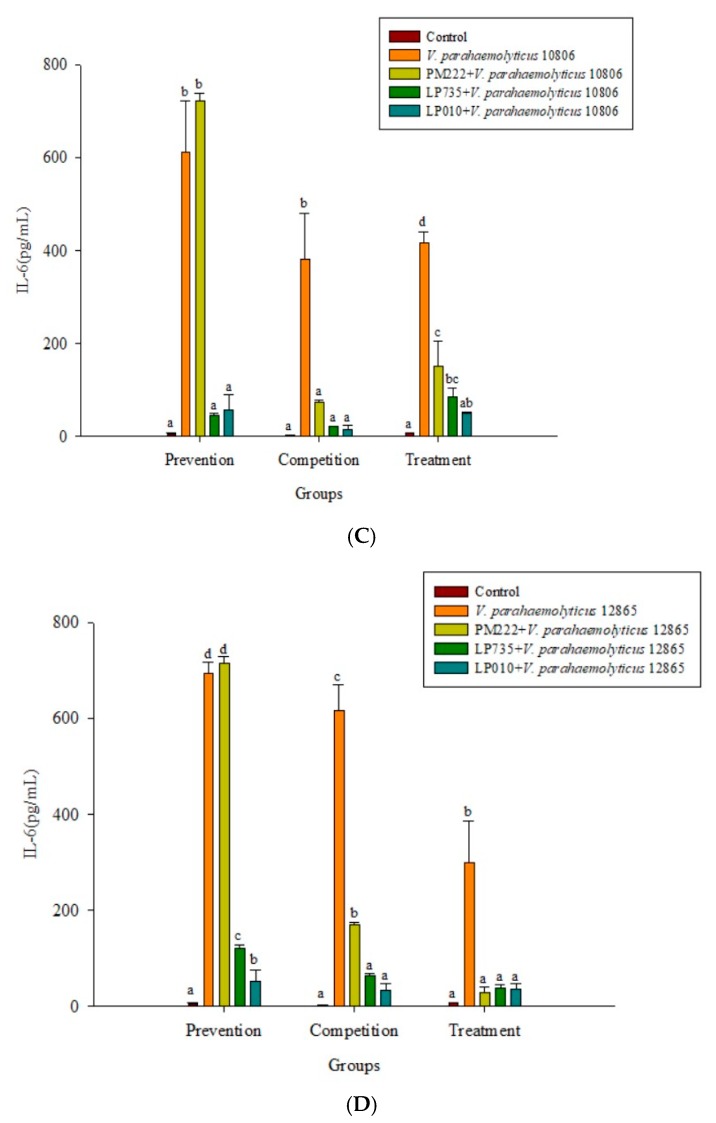
Inhibition of *Vibrio parahaemolyticus* (**A**) BCRC 10806, (**B**) BCRC 12865–induced TNF-α synthesis, (**C**) BCRC 10806, and (**D**) BCRC 12865–induced IL-6 synthesis in RAW 264.7 cell line by different lactic acid bacteria. ^a,b,c,d^ Values in the same group with different superscripts mean a significant difference (*p* < 0.05) using the Duncan’s multiple range test. Prevention: LAB (10^7^ CFU/mL) was added for two hours followed by *V. parahaemolyticus* (10^5^ CFU/mL) for 15 h. Competition: LAB and *V. parahaemolyticus* were added simultaneously and incubated for 17 h. Treatment: *V. parahaemolyticus* was added for two hours before adding LAB for 15 h.

**Figure 3:**
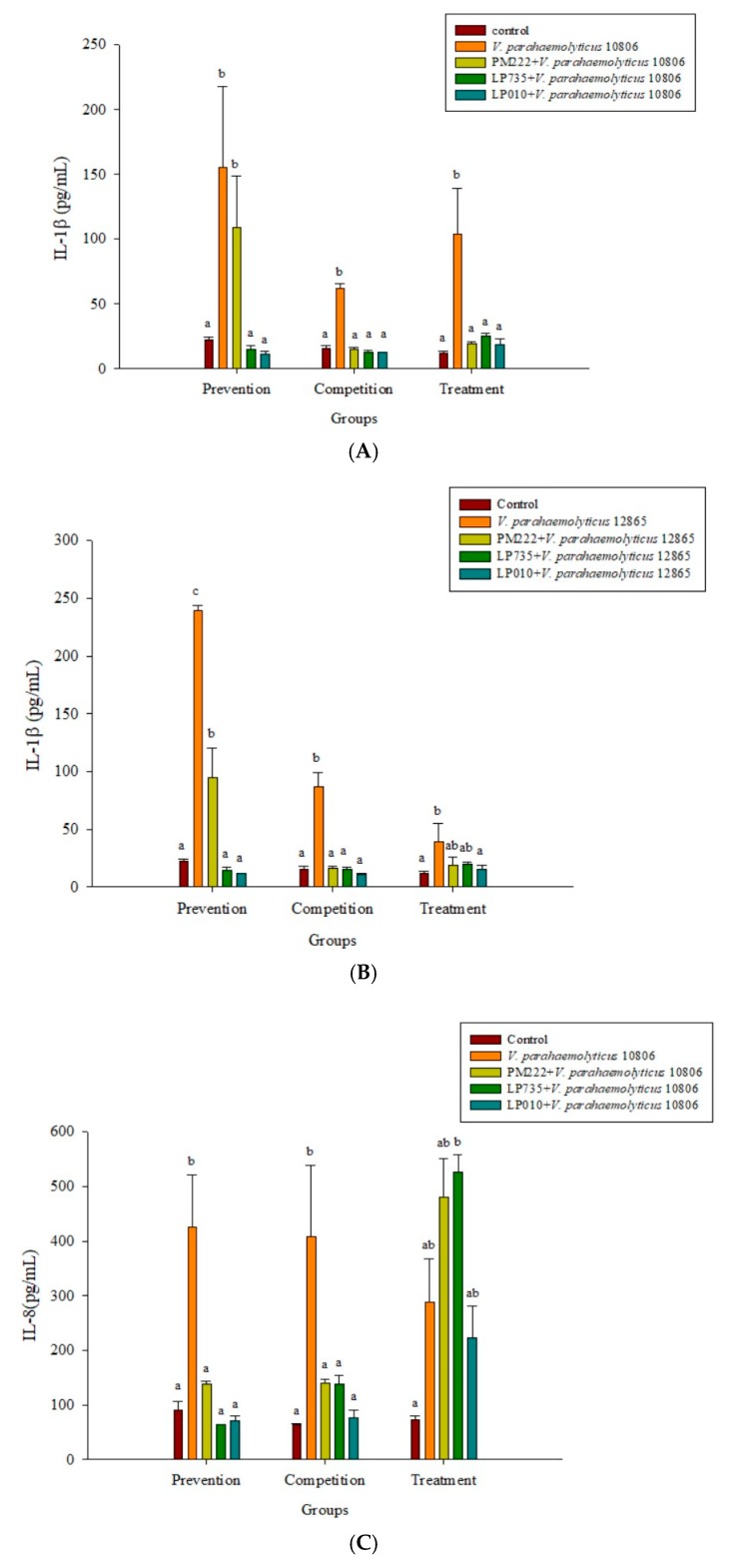

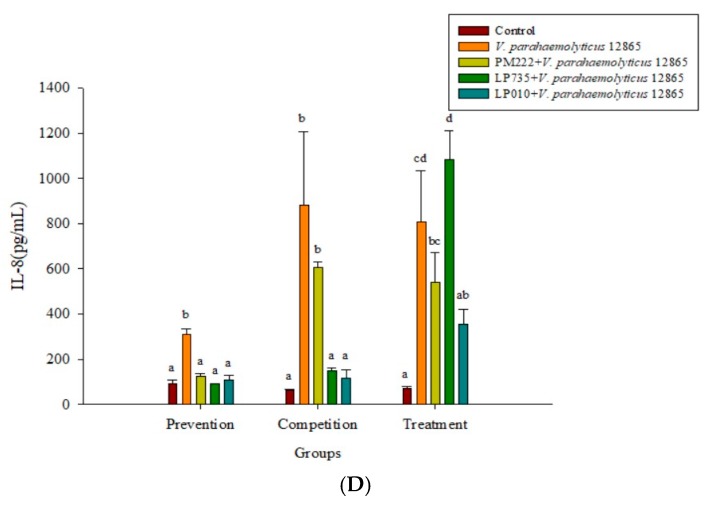
Inhibition of *Vibrio parahaemolyticus* by (**A**) BCRC 10806 and (**B**) BCRC 12865–induced IL-1β synthesis in RAW 264.7 cell line; (**C**) BCRC 10806 and (**D**) BCRC 12865–induced IL-8 synthesis in the HT-29 cell line by different lactic acid bacteria. ^a,b,c^ Values in the same group with different superscripts mean significant difference (*p* < 0.05) using the Duncan’s multiple range test. Prevention: LAB (10^7^ CFU/mL) was added for two hours followed by *V. parahaemolyticus* (10^5^ CFU/mL) for 15 h. Competition: LAB and *V. parahaemolyticus* were added simultaneously and incubated for 17 h. Treatment: *V. parahaemolyticus* was added for two hours before adding LAB for 15 h.

As shown in Figure 2A,B, three strains of lactobacilli in both the prophylaxis group and the concurrent group effectively reduced the secretion of TNF-α in RAW 264.7 macrophages in *V. parahaemolyticus* BCRC 10806 and BCRC 12865. No significant difference was found between the treatment group and the *V. parahaemolyticus* group, probably because LAB did not colonize the cells to protect the cells, resulting in cell damage *V. parahaemolyticus*. As shown in Figure 2C,D, three strains of LAB in the treatment group and the simultaneous treatment group significantly inhibited the secretion of IL-6 by RAW 264.7 cells induced by *V. parahaemolyticus* BCRC 10806 and 12865. As shown in Figure 3A,B, the three strains of LAB could significantly inhibit IL-1β secretion in *V. parahaemolyticus* BCRC 10806 and BCRC 12865-induced IL-1β cytokine secretion by RAW 264.7 macrophages in the prophylaxis group. The treatment group and the concurrent group LP 735 and LP 010 strains had a more potent inhibitory effect on IL-1β expression in the prophylaxis group than the PM 222 strain. As shown in Figure 3C,D, the three strains of LAB significantly inhibited the increase in IL-8 normally induced by *V. parahaemolyticus* BCRC 10806 and BCRC 12865 in the prophylaxis group and concurrent group of HT-29 intestinal epithelial cells. These results indicate that *Lactobacillus* colonization in the gut can protect the host from *V. parahaemolyticus*-induced injury by reducing the production of proinflammatory factors.

### 2.4. Effects of Adding LAB and V. parahaemolyticus on the Expression of Inflammatory-Related Genes in Different Cell Lines

**Figure 4:**
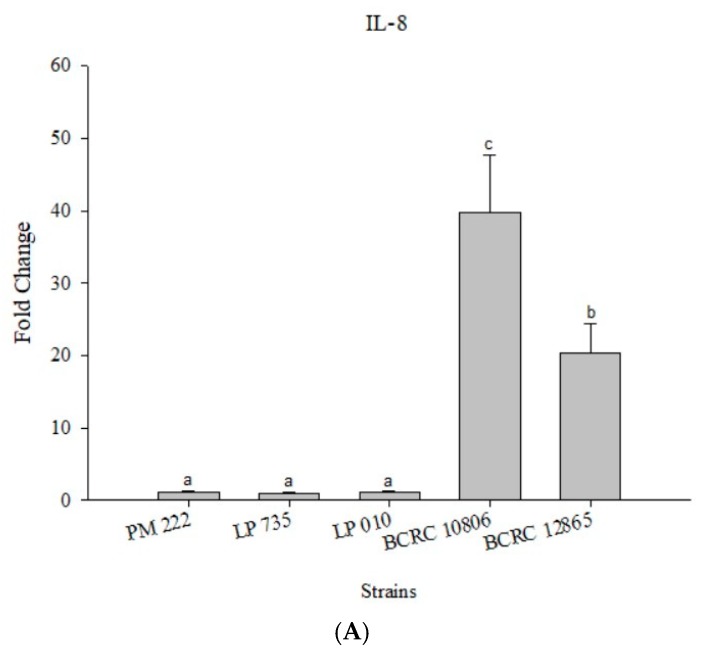

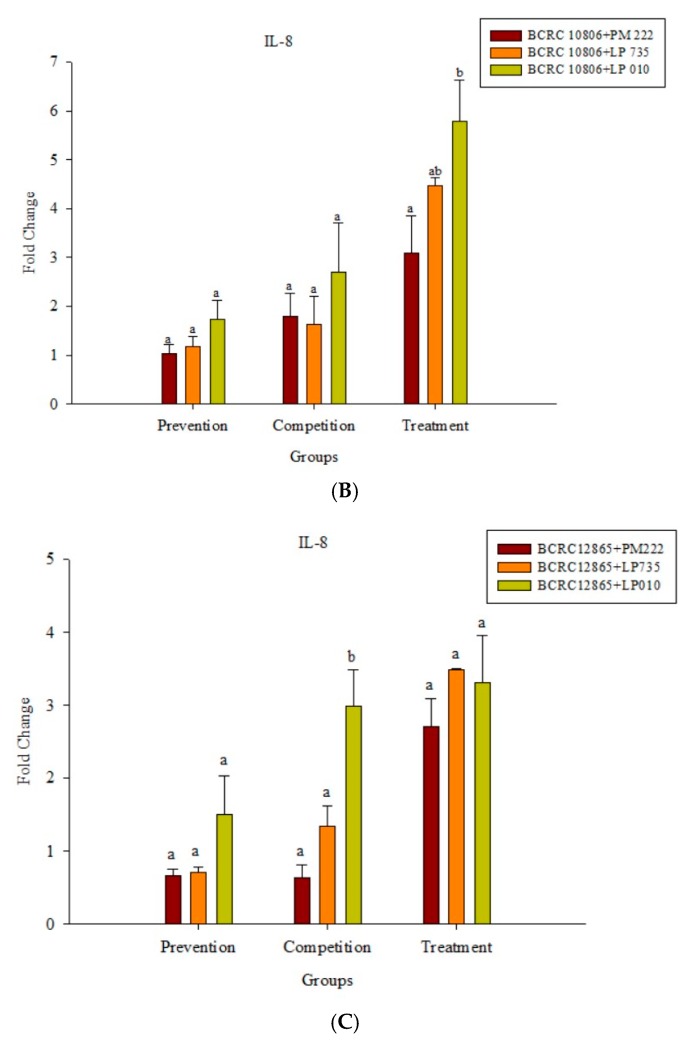
(**A**) Real-time polymerase chain reaction (PCR) analysis of Caco-2 intestinal epithelial cells stimulated with lactic acid bacteria and *Vibrio parahaemolyticus* to observe the change of gene expression in IL-8 mRNA, with three different models using lactic acid bacteria, (**B**) BCRC 10806, and (**C**) BCRC 12865 to stimulate Caco-2 intestinal epithelial cells to observe the change in gene expression of IL-8 mRNA. Expression of target genes (IL-8) was normalized to β-actin and is presented as mean ± standard error. ^a,b,c^ values in the same group with different superscripts mean significant difference (*p* < 0.05) using the Duncan’s multiple range test.

According to the results presented in Figure 4A, LAB did not induce IL-8 expression in Caco-2 cells, but the expression of IL-8 significantly increased upon treatment with *V. parahaemolyticus*, and treatment with strain BCRC 10806 induced the highest amount of IL-8. The results presented in Figure 4B,C show that the three LAB strains all have an inhibitory effect on the inflammatory response to IL-8 against *V. parahaemolyticus* BCRC 10806 and BCRC 12865 in three different modes compared with *V. parahaemolyticus* alone.

**Figure 5:**
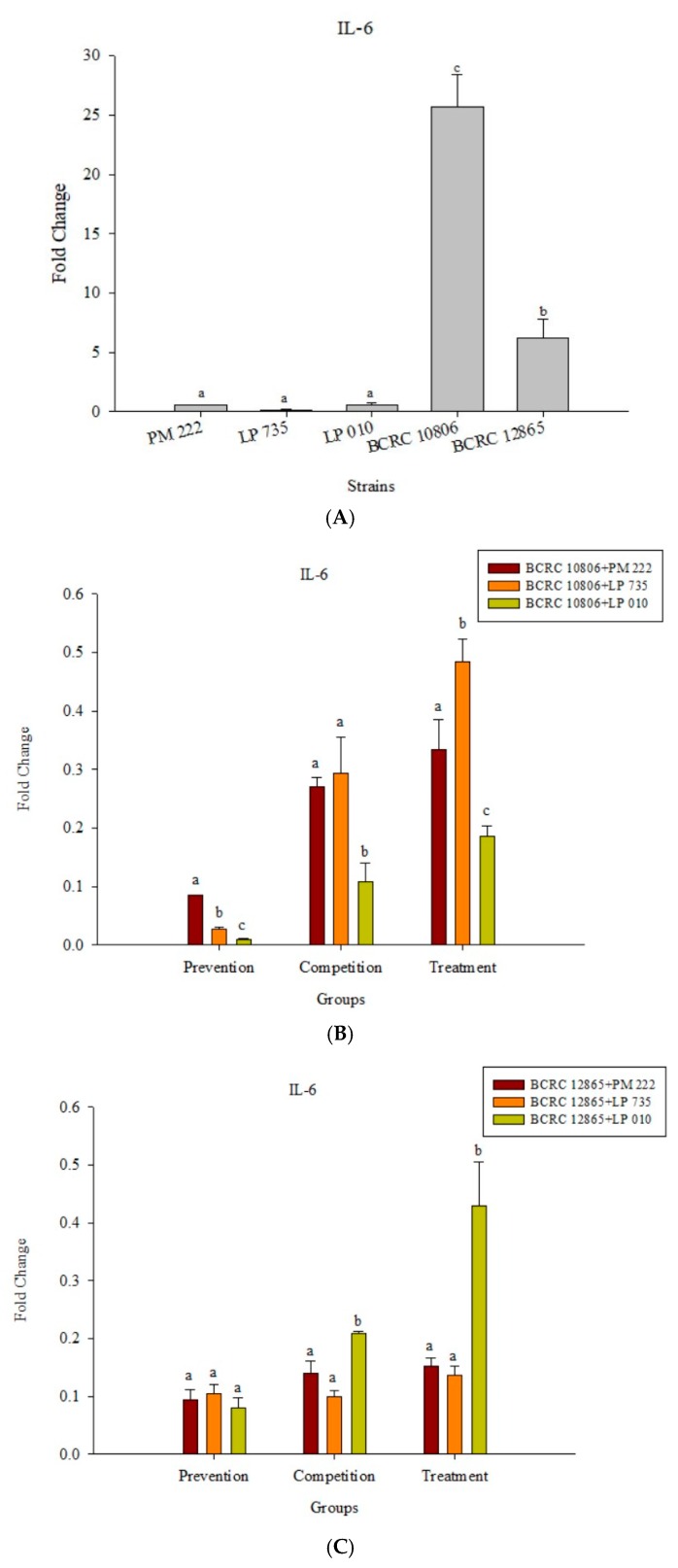
(**A**) Real-time PCR analysis of Raw 264.7 macrophage cell line stimulated with lactic acid bacteria and *Vibrio parahaemolyticus* to observe the change in gene expression of IL-6 mRNA, with three different models using lactic acid bacteria, (**B**) BCRC 10806, and (**C**) BCRC 12865 to stimulate the Raw 264.7 macrophage cell line to observe the change in gene expression of IL-6 mRNA. The expression of target genes (IL-6) was normalized to β-actin and is presented as mean ± standard error. ^a,b,c^ Values in the same group with different superscripts mean significant difference (*p* < 0.05) using the Duncan’s multiple range test.

**Figure 6:**
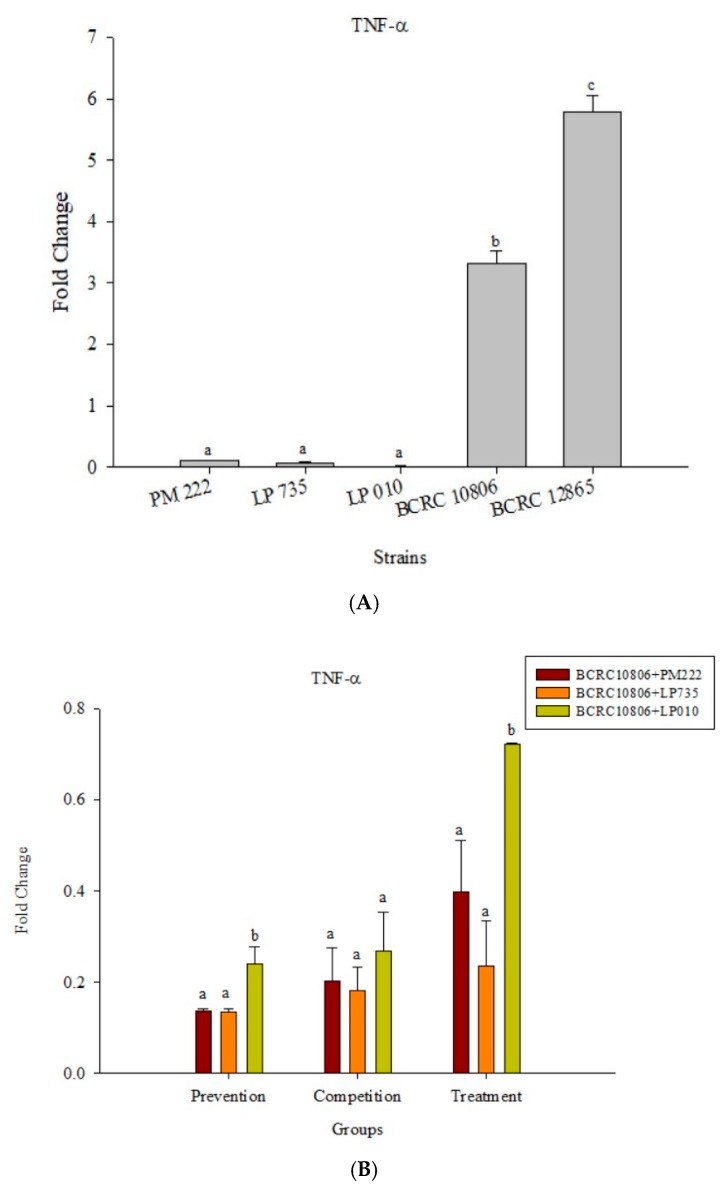

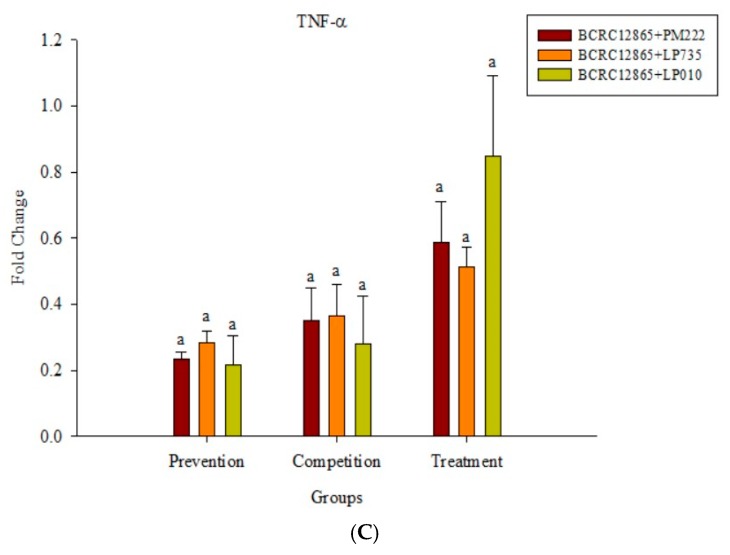
(**A**) Real-time PCR analysis of the Raw 264.7 macrophage cell line stimulated with lactic acid bacteria and *Vibrio parahaemolyticus* to observe the change in gene expression of TNF-α mRNA, with three different models using lactic acid bacteria, (**B**) BCRC 10806, and (**C**) BCRC 12865 to stimulate the Raw 264.7 macrophage cell line to observe the change in gene expression of TNF-α mRNA. The expression of target genes (TNF-α) was normalized to β-actin and is presented as mean ± the standard error. ^a,b,c^ Values in the same group with different superscripts mean significant difference (*p* < 0.05) using the Duncan’s multiple range test.

The results presented in Figure 5A show that LAB induced low levels of IL-6 expression in Raw 264.7 macrophages but induced significantly higher IL-6 performance relative to the *V. parahaemolyticus* BCRC 12865 group and LAB in the prevention group. The role of both groups and the treatment group effectively inhibited IL-6 performance (Figure 5B,C). The results presented in Figure 6A show that LAB do not induce Raw 264.7 to express TNF-α. However, upon treatment with *V. parahaemolyticus* BCRC 12865 and BCRC 10806, TNF-α activity significantly reduced compared with that of IL-6 (Figure 6B,C).

**Figure 7:**
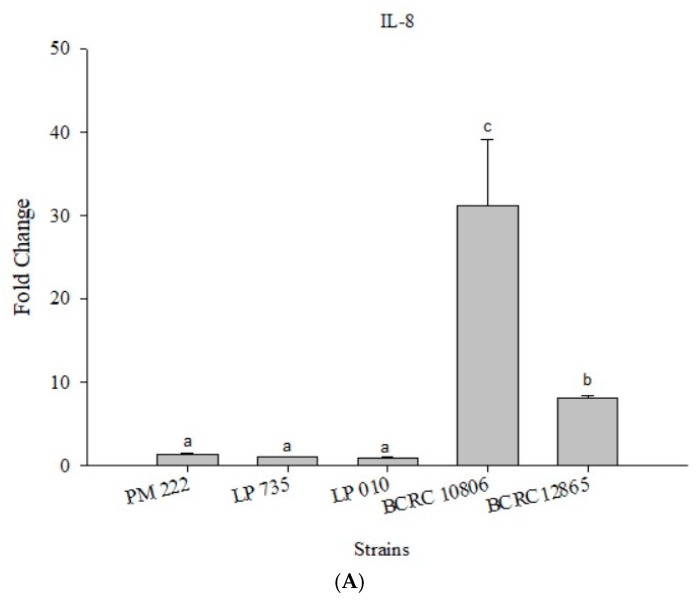

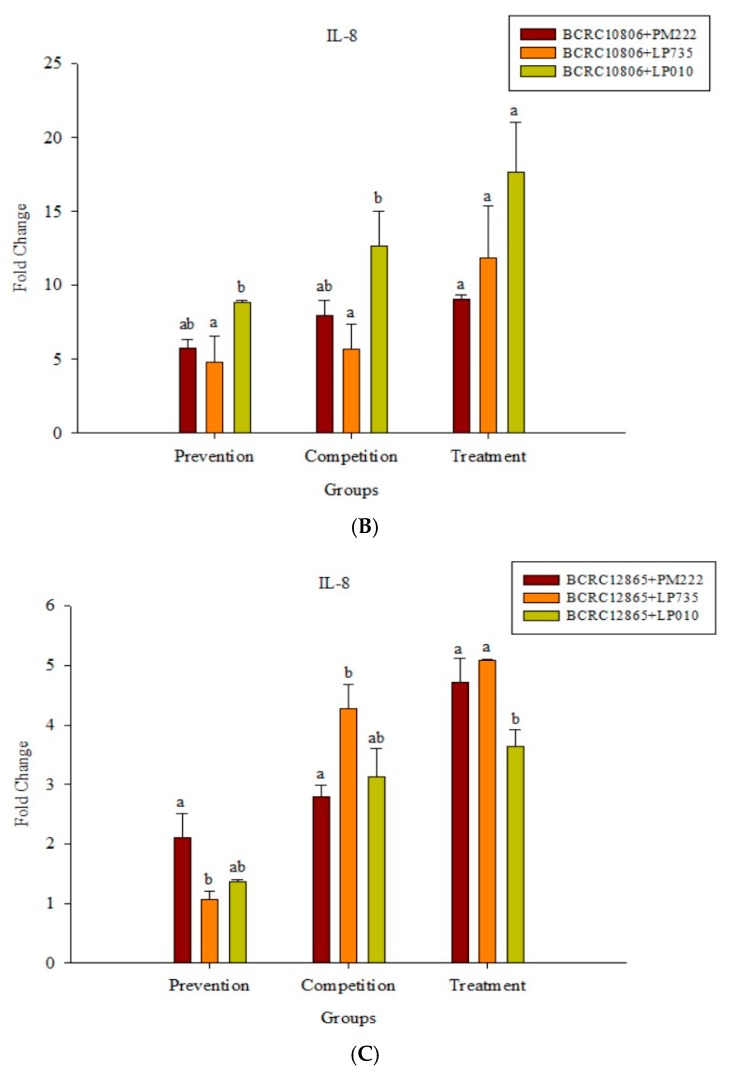
(**A**) Real-time PCR analysis of human colorectal adenocarcinoma cells HT-29 stimulated with lactic acid bacteria and *Vibrio parahaemolyticus* to observe the change in gene expression of IL-8 mRNA, with three different models using lactic acid bacteria, (**B**) BCRC 10806, and (**C**) BCRC 12865 to stimulate human colorectal adenocarcinoma cells HT-29 to observe the change in gene expression of IL-8 mRNA. The expression of target genes (IL-8) was normalized to β-actin and is presented as mean ± standard error. ^a,b,c^ Values in the same group with different superscripts mean significant difference (*p* < 0.05) using the Duncan’s multiple range test.

As shown in Figure 7A, IL-8 expression was very low in the HT-29 colorectal cancer cell line after treatment with LAB. In addition, two strains of *V. parahaemolyticus* were found to promote the expression of IL-8, but the expression of IL-8 was significantly higher in BCRC 10806-treated cells. All three strains of LAB were effective in the prophylaxis group, the treatment group, and the competition group compared with the group treated with *V. parahaemolyticus* alone (Figure 7B,C).

### 2.5. Adsorption Experiments on Mouse Epithelial Cells and Intestinal Mucus

Three strains of *Lactobacillus* had different degrees of adhesion to mouse intestinal epithelial cells and mucus. Our results showed that *L. plantarum* PM 222 and LP 010 had strongly adhered on intestinal epithelial cells (26.4 and 18.7 bacteria/cell, respectively) and adhered highly to mucus [Optical density (OD) 570 nm of 0.12 and 0.09, respectively] (Table 3). On the contrary, *Lactobacillus plantarum* LP 735 showed lower adhesion to both intestinal epithelium (14 bacteria/cell) and intestinal mucus (OD570 nm of 0.05).

### 2.6. Effect of LAB on Serum Immunity of V. parahaemolyticus in Mice

**Figure 8:**
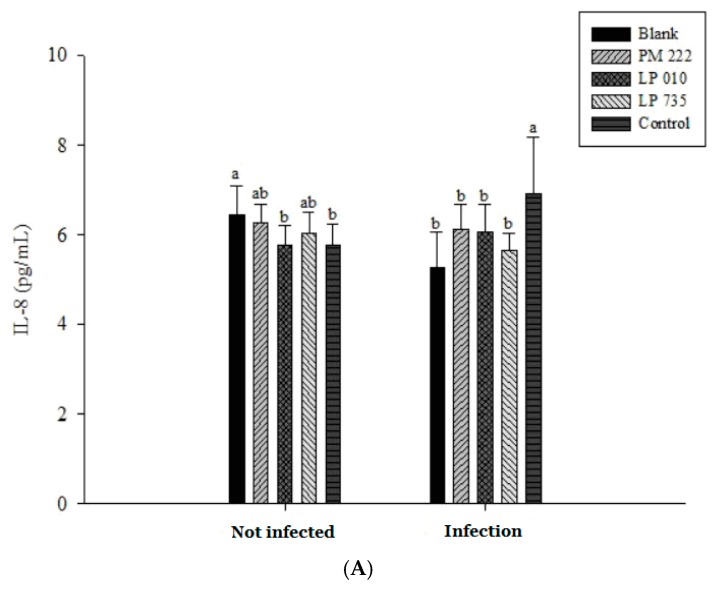

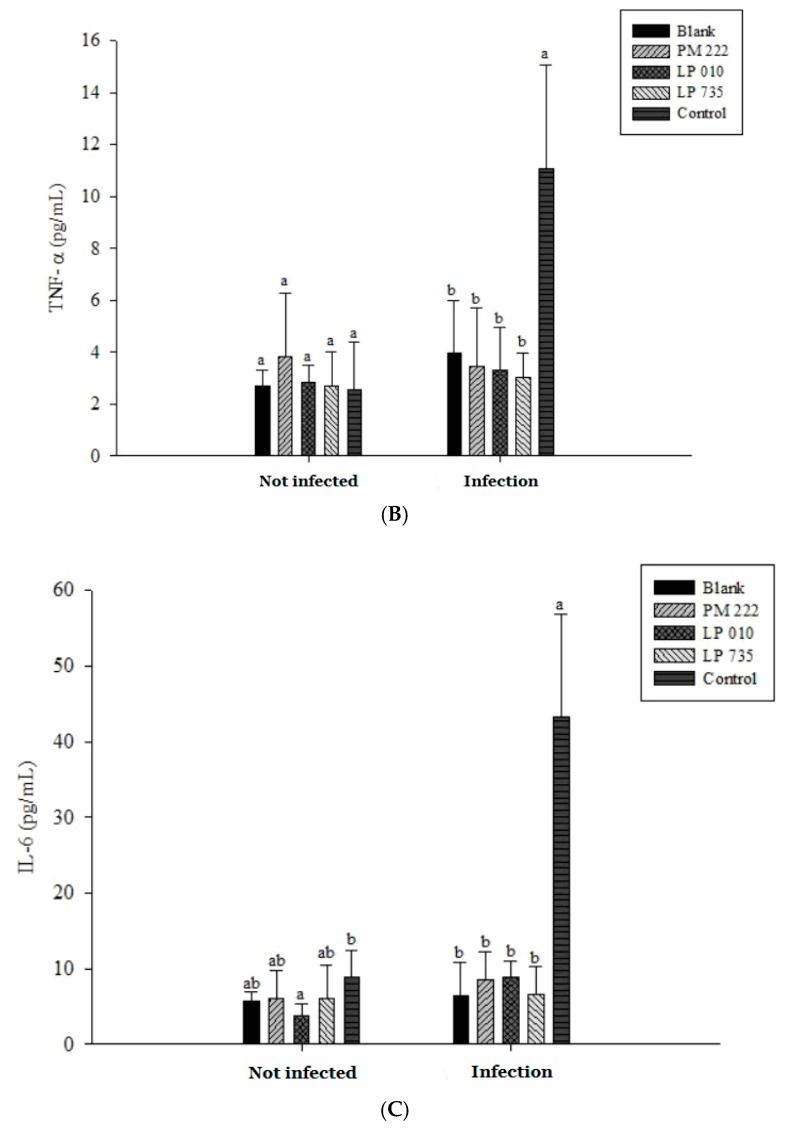
Mice were fed *Vibrio parahaemolyticus* BCRC 12865, and the expression of (**A**) IL-8, (**B**) TNF- α, and (**C**) IL-6 in serum was observed after 24 h. Each value is expressed as mean ± standard deviation (*n* = 8). ^a,b^ Values in the same group with different superscripts mean significant difference (*p* < 0.05) using the Duncan’s multiple range test.

The results of IL-8 in serum showed that the group given *V. parahaemolyticus* alone was significantly different from the group treated with LAB (Figure 8A). However, IL-8 expression remained low in both instances, presumably because the *V. parahaemolyticus* infection was not sufficiently long to elicit a response. The amount of TNF-α expression can be seen in Figure 8B. TNF-α and IL-6 expression increased significantly in the *V. parahaemolyticus*-only treated group compared with that of the LAB group when continuously administered for seven days (Figure 8B,C). Therefore, we inferred that secretion of TNF-α and IL-6 by mice fed *V. parahaemolyticus* caused an inflammatory reaction.

### 2.7. Gut Histology Changes after Treatment with V. parahaemolyticus

**Figure 9:**
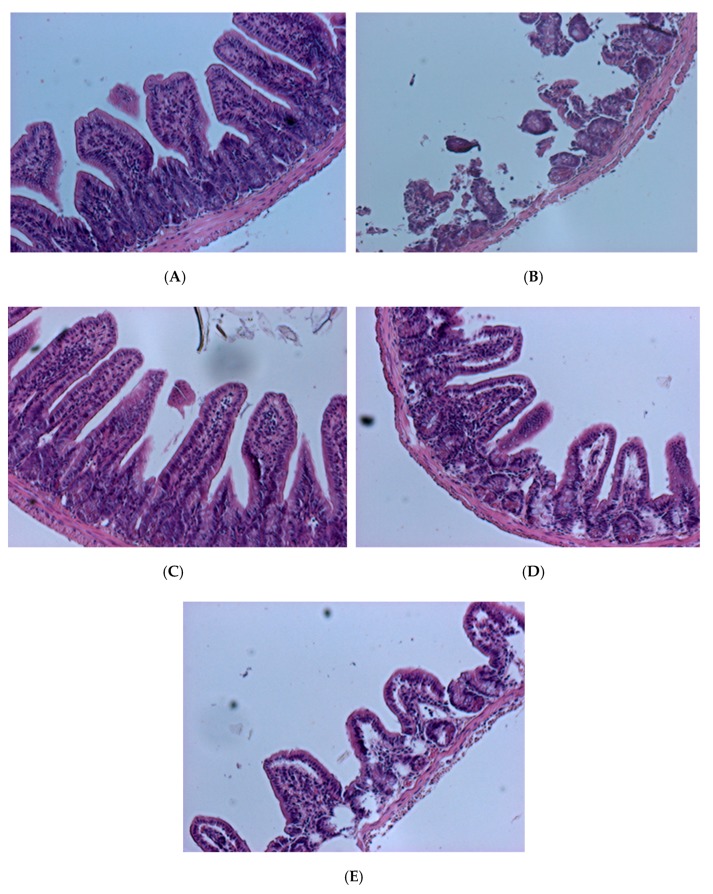
Histopathological changes to the mouse intestine 24 h post-challenge with *Vibrio parahaemolyticus* BCRC 12865. The images of myocardial architecture were magnified 200 times. (**A**) Blank group, (**B**) Control, (**C**) PM 222 + BCRC 12865, (**D**) LP 010 + BCRC 12865 and (**E**) LP 735 + BCRC 12865.

**Figure 10:**
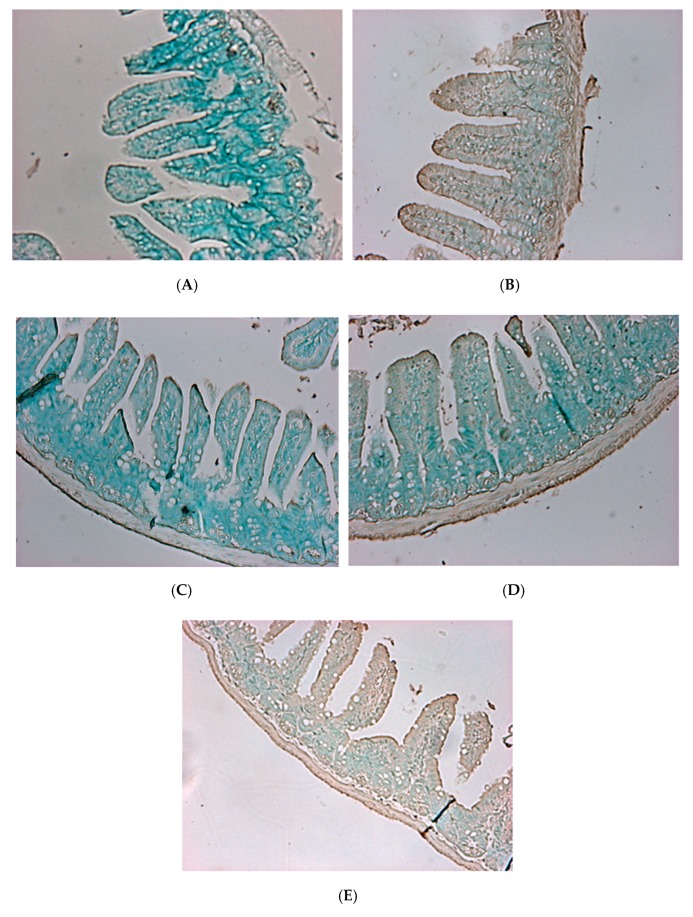
Histopathological changes to the mouse intestine 24 h post-challenge with *Vibrio parahaemolyticus* BCRC 12865. The images of myocardial architecture were magnified 200 times. (**A**) Blank group, (**B**) Control, (**C**) PM 222 + BCRC 12865, (**D**) LP 010 + BCRC 12865 and (**E**) LP 735 + BCRC 12865.

To confirm the degree of intestinal tissue damage caused by *V. parahaemolyticus*, tissue sections and Hematoxylin and eosin staining were performed (Figure 9 and Figure 10). Histological results showed that in the blank group of mice (fed PBS (phosphate buffer solution), not given *V. parahaemolyticus*), intestinal villi were long, slim, and regularly arranged with a normal appearance and pronounced structure (Figure 9A). Small intestine villi were severely damaged, and their integrity was lost in the control mice that were tube-fed with phosphate buffer solution (PBS) containing 10^9^ CFU/mL *V. parahaemolyticus* BCRC 12865 24 h prior to sacrifice (Figure 9B). Mice fed the control *L. plantarum* PM 222 or LP 010 strains showed decreased villus injury in varying degrees after feeding with *V. parahaemolyticus* (Figure 9C,D). In mice treated with *L. plantarum* strain LP735, the villi were shortened, crowded, and irregularly arranged compared with the blank group (Figure 9E).

Apoptosis staining showed that almost no apoptosis occurred in the blank group mice (Figure 10A), whereas the control group (Figure 10B) showed extensive apoptosis. However, the *L. plantarum* PM 222- and LP 010-fed groups (Figure 10C,D) showed a small amount of apoptosis. The LP 735 group (Figure 10E) also had obvious apoptosis compared with the blank group, and the enteritis arc Hematoxylin and eosin-stained sections of the *L. plantarum* strains PM 222 and LP 010 in both strains of the BCRC 12865 group showed that the villi retained their integrity, showed less damage, and showed only a small amount of apoptosis. These results indicate that these two strains can protect mice against *Vibrio* spp. infection. According to experiments with the mouse intestinal mucosa and intestinal epithelial cells, LAB PM 222 were the most efficiently adsorbed. In the intestinal histology, PM 222 was also observed to have the best protective effect, proving that a strong adsorption capacity in LAB can reduce *V. parahaemolyticus*-induced damage.

## 3. Materials and Methods

### 3.1. LAB Strain and Cell Line Culture

*L. plantarum* PM 222, LP 010, and LP 735 strains isolated from fermented vegetables and identification with an API 50 CHL kit (La Balme Les Grottes, Montalien, Jeraen, France were cultured in *Lactobacilli* MRS (de Man, Rogosa and Sharpe) Broth with 0.05% l-cysteine. *V. parahaemolyticus* BCRC 10806 and BCRC 12865 were cultured in TSB (Tryptone Soy Broth). NaCl (2.5%) was added to the culture medium, and both LAB and *V. parahaemolyticus* strains were incubated at 37 °C for 18 h.

The Caco-2 cell line was grown in Dulbecco’s Modified Eagle Medium (DMEM). HT29 and Raw 264.7 cell lines were cultured in MEM (Minimum Essential Medium) supplemented with 10% FBS and sodium bicarbonate (NaHCO_3_). The cell cryostat was moved from the liquid nitrogen barrel to a 37 °C water bath for rapid thawing. Cell suspensions were thawed in a 75 T Cell Culture Flask or cell culture dish, and a sterile pipette was used to add the appropriate amount of cell culture medium. Cells were incubated at 37 °C at 5% carbon dioxide (CO_2_).

### 3.2. Cell Viability Analysis

Cell viability in response to *V. parahaemolyticus* and *L. plantarum* was determined following the methods described in Fernández et al. [11] with modifications. Caco-2 cells were seeded into a 24-well plate at 2 × 10^5^ cells/mL per well. After culturing for 24 h, the cell culture fluid was aspirated off, and cells were washed twice with PBS buffer solution. MTT assays, in response to *V. parahaemolyticus* (10^5^ CFU/mL) and *L. plantarum* (10^7^ CFU/mL), were performed at 2 and 4 h, respectively.

### 3.3. LAB Inhibits V. parahaemolyticus in Food Mode

*V. parahaemolyticus* was detected using the National Standards of Republic of China CNS 12358, N6210 (Food Microbiological Test—*V. parahaemolyticus*) methods and Montiel et al. [12]. Samples of snapdragon snapper were purchased at a shopping mall. Each sample weighed approximately 400 g. The surface of the film was irradiated with an ultraviolet (UV) lamp for 30 min. The bream fillets were cut into 20 g pieces. The thickness of each piece was kept as consistent as possible to ensure experimental accuracy. Inhibition experiments were conducted by adding a 1:100 ratio of *V. parahaemolyticus* (10^5^ CFU/mL) and LAB (10^7^ CFU/mL). LAB supernatant or LAB was added, and the samples were incubated at 4 °C or room temperature for 1 and 4 h; then, a 180-mL diluted solution PBS added by the stomach was patted for 2 min, serially diluted, and then poured into a petri dish with thiosulfate citrate bile salts (TCBS) sucrose agar. The colonies were incubated overnight at 37 °C, and colonies of *V. parahaemolyticus* grown in TCBS agar for identification medium was blue to green in color.

### 3.4. Levels of IL-1β, IL-6, IL-8, and TNF-α Cytokines Measured by ELISA

The methods of Gueimonde et al. [13] and Satish Kumar et al. [14] were used with some modifications. LAB (1 mL; 10^9^ CFU/mL) and *V. parahaemolyticus* (10^8^ CFU/mL) were each centrifuged at 10,000 rpm for 10 min. The supernatant was discarded, washed twice, and then dissolved in 1 mL of cell culture medium and diluted to the required number of experimental bacteria for later use.

To observe the integrity of the HT-29 and RAW 264.7 cell lines in the 75 T Cell Culture Flask, the cells were cultured in the shape of an angled petri dish. Cells were detached from the petri dish with trypsin-EDTA (Ethylenediaminetetraacetic acid) or a cell scraper. Cell count was determined with a hemocytometer, and the cell concentration was adjusted to 3 × 10^5^ and 2 × 10^5^ cells/mL. Cells were added to a 24-well plate, uniformly mixed, and incubated at 37 °C and 5% CO_2_ for 48 h. After confirmation of complete cell attachment, the old medium was aspirated and washed twice with PBS. The experiment was conducted in 3 experimental modes: (1) Prevention: LAB (10^7^ CFU/mL) was added for 2 h followed by *V. parahaemolyticus* (10^5^ CFU/mL) for 15 h; (2) Competition: LAB and *V. parahaemolyticus* were added simultaneously and incubated for 17 h; and (3) Treatment: *V. parahaemolyticus* was added for 2 h before adding LAB for 15 h. After the incubation time, the cell supernatant was collected and stored at −20 °C until use.

The cell cultures of the above experiments were collected and modified according to the methods of Lian et al. [15] and Shimohata et al. [1]. IL-8, IL-6, IL-1β, and TNF-α concentrations were determined by ELISA. The ELISA was performed using the Becton, Dickinson and Company (BD) Biosciences Kit according to the manufacturer’s instructions.

### 3.5. Total RNA Extraction

RNA extraction was performed according to the methods of Matlawska-Wasowska et al. [16] and Shimohata et al. [1]. Caco-2, Raw 264.7, and HT-29 cells were inoculated at 2.5 × 10^5^, 3.5 × 10^5^, and 4 × 10^6^ CFU/mL into 10-cm cell culture dishes and cultured for 24 to 48 h. Then, LAB (10^7^ CFU/mL) and *V. parahaemolyticus* (10^5^ CFU/mL) were added for 11.5–15 h, respectively. After incubation, cells were washed twice with PBS, and PBS was aspirated completely before 1 mL of TRIzol solution was added to lyse the cells. After the cells were fully lysed, lyzed cells were added to a 1.5-mL microcentrifuge tube using a cell scraper. A total of 400 μL of chloroform was added, and the sample was mixed for 10 min. The tubes were centrifuged at 12,000 rpm for 15 min at 4 °C. After centrifugation, 2–300 μL of supernatant was carefully drained into a new microcentrifuge tube. The DNA extracted into the middle layer was centrifuged once more. The supernatant was added to 500 mL of isopropyl alcohol (isopropanol), gently mixed, and allowed to stand for 10 min before being centrifuged at 12,000 rpm for 15 min at 4 °C. The supernatant was removed, washed with 1 mL of 70–75% alcohol, and centrifuged. Any remaining supernatant was removed, and the pelleted sampled was placed in an exhaust hood to dry. Finally, 50 μL of diethyl pyrocarbonate (DEPC)-treated water was heated to 65 °C for 5 min; the RNA was fully dissolved with DEPC water, Deoxyribonuclease I was added to remove DNA in the sample, and the final RNA sample was stored at −80 °C.

### 3.6. Real-Time Quantitative Polymerase Chain Reaction

Using a SuperScript^®^ (Invitrogen, Carlsbad, CA, USA) III First-Strand Synthesis SuperMix for real-time quantitative PCR (qRT-PCR), 1 g of total RNA was weighed into a PCR tube, and 10 μL 2X RT Reaction Mix and 2 μL RT Enzyme Mix were added to make up to a total volume of 20 μL. Subsequently, the PCR tubes were heated at 25 °C for 10 min, 50 °C for 30 min, and then 85 °C for 5 min to terminate the reaction. Finally, 1 μL (2 U) of *E. coli* RNase H was added and reacted at 37 °C for 20 min to remove excess RNA. The synthetic cDNA was stored at −20 °C.

Gene expression analysis was based on a comparison with the housekeeping gene β-actin as a control, and the fold change was calculated by a previously described formula [17]. With the KAPATMSYBR^®^ (Roche, Woburn, MA, USA) FAST qPCR Kit, the total PCR volume was 20 μL. DEPC water was added to the first 8 rows, and then 0.4 μL of Forward Primer (10 μM), 0.4 μL of Reverse Primer (10 μM), 10 μL of KAPATMSYBR^®^ FAST qPCR Master mix (2X) Universal, 0.4 μL of ROX LOW, and 2 μL of cDNA were added and into the ABI7500 system real-time PCR machine. Taking β-actin as an example, the reaction conditions were as follows: 94 °C for 5 min, 40 cycles at 94 °C for 30 s, 57 °C for 1 min, and 72 °C for 40 s. To confirm that the PCR product was a specific product, the dissociation curve mode was used: 95 °C for 15 s, 60 °C for 1 min, and 95 °C for 15 s.

Real-time quantitative PCR data were analyzed using the 2^−ΔΔCt^ method, which Livak and Schmittgen used in 2001 [18]. The formula is as follows:

Fold Change = 2^Ct target (control) − Ct target (treatment)/2Ct β-actin (control) − Ct β-actin (treatment)^

The fold change of the formula is the relative performance of the test gene mRNA in the experimental group calculated as the housekeeping control gene (β-actin).

### 3.7. LAB Treatment to Inhibit V. parahaemolyticus Infection in Animal Model

Forty male BALB/c five-week-old mice were purchased from Bio Lasco, Taiwan Co., Ltd., Taipei, Taiwan and were housed in individually ventilated cages (IVC). The litter was fed for one week with Lignocel FS14 (Rettenmaier & Söhne, Rosenberg, Germany). The feed was given to Altromin 1324 (Altromin GmbH, Lage, Germany) after weight distribution to each group, at the age of 6 weeks of age to start the experiment.

Viral BCRC 12865 was modified according to Yang et al. [19] to investigate whether it affects gut and immune responses in mice. Mice were divided into 5 groups with 8 mice in each group, which were fed with (1) *L. plantarum* PM 222 (10^8^ CFU/day), (2) *L. plantarum* LP 010 (10^8^ CFU/day), or (3) *L. plantarum* LP 735 (10^8^ CFU/day) for 7 consecutive days. The blank control group (4) and control group (5) were fed with PBS. On the 8th day, 200 μL of *V. parahaemolyticus* BCRC 12865 (10^9^ CFU/day) was fed to the mice in the control group and the experimental group. The blank control group was only fed 200 μL of PBS. After the administration of *V. parahaemolyticus* for 24 h, mice were sacrificed, and intestinal and blood samples were collected.

The experiment was performed according the method described by Yang et al. [19]. Mouse small intestine cells were detached using mesh and collected into 5 mL DMEM. The suspension was washed twice by centrifugation at 100 rcf for 10 min. After 1 mL of LAB (10^9^ CFU/mL) and 1 mL of epithelial cells were mixed, they were incubated at 37 °C for 1.5 h. Bacteria-bound epithelial cells were centrifuged at 100× *g* for 10 min and washed twice with PBS. Cells were applied to a slide for Gram staining. LAB counts on epithelial cells were performed using an optical microscope at 400× magnification.

The inner surface of the mouse small intestine (duodenum, jejunum, and part of the ileum) was isolated using mesh, and the mucus was collected in 250 μL of ice-cold PBS. The resulting suspension was mixed and centrifuged at 12,000 rcf for 10 min to remove cell debris and bacteria. The supernatant was collected, the protein concentration was measured using a UV spectrophotometer, and the absorbance was measured at 280 nm. Mucus extract (150 μL; 0.5 mg protein/mL) was added to a 96-well plate and incubated overnight at 4 °C. After overnight incubation, the cells were washed with PBS, and 100 μL (10^9^ CFU/mL) of LAB and PBS were added and incubated for 2 h at 20 °C. After culturing, cells were washed 3 times with PBS. The adherent bacteria were fixed at 60 °C for 20 min and stained with 100 μL of filtered 0.5% crystal violet solution for 45 min. After staining, cells were washed with PBS 5 times and treated with 100 μL of citrate buffer (20 Mm, pH 4.3) for 45 min at room temperature. Absorbance was measured with a microplate spectrometer at a 570 nm wavelength. The average absorbance value indicates the ability of the strain to adsorb mucus.

Tissue sections were stained with hematoxylin and eosin (H & E) and observed under a microscope. First, the sliced specimen was desensitized, soaked in hematoxylin solution for 3 min, and washed with ddH_2_O. Then, the sample was soaked in 85% alcohol for 1 min, eosin solution for 5 min, and then in 70%, 80%, and 90% alcohol, and finally 100% ethanol was added for 2 min. The xylene solution was soaked for 30 s (twice), then dried and sealed [20,21]. The occurrence of apoptosis was confirmed by TUNEL (Terminal deoxynucleotidyl transferase dUTP nick end labeling) staining. Apoptosis staining procedures were performed as described in the Apo-BrdU-IHCTM in situ DNA fragmentation assay kit (catalog no. K403-50; BioVision, Mountain View, CA, USA) for staining to further observe apoptosis of the tissue cells.

### 3.8. Statistical Analysis

Data were statistically analyzed using the Statistical Product and Service Solutions version 20.0. The values are expressed as means ± standard deviation. One-way analysis of variance (one-way ANOVA) was used to confirm differences within the experimental group. Duncan’s New Multiple Range Test was used to analyze the differences between the means of the experimental groups. *p* < 0.05 was considered significant.

## 4. Discussion

Fernández et al. reported that *V. parahaemolyticus* was cytotoxic to epithelial cells in a time-dependent manner, with no cell lysis occurring at two hours, but an increasing amount of cell lysis was observed three or four hours later [11]. When Caco-2 cells were co-cultured with *V. parahaemolyticus* for four hours, the cell number decreased because of cytotoxicity caused by *V. parahaemolyticus* [11]. Chiu et al. found that *L. plantarum* co-cultured with Caco-2 cells did not affect cell viability within 10 h, but cell viability decreased significantly between 12 and 14 h [22].

Mahmoud et al. found that *V. parahaemolyticus* at 5.9 log CFU/g inoculated with 150 mg/mL lactic acid inhibited the growth of enteroviruses compared with 300 mg/mL citric acid, which was completely inhibited, indicating the effectiveness of lactic acid against *V. parahaemolyticus* [23]. Mahmoud indicated that lactic acid and citric acid wash solutions could offer an inexpensive, natural, and effective approach to control *V. parahaemolyticus* in sterilized shucked oysters for the oyster industry [23].

The benefits, roles, and mechanisms of probiotics in the host have not yet been fully elucidated, but some of the known probiotic species, such as *L. rhamnosus*, *L. acidophilus*, and *L. plantarum*, were reported to benefit host health [24]. Boonma et al. found that *L. rhamnosus* L34 and *L. casei* L39 inhibited *Clostridium difficile*-induced IL-8 in colonic epithelial cells HT-29 [25]. Kim et al. compared the differences between live *L. acidophilus* (10^9^ and 10^10^ CFU/mL) and thermo-killed LAB (10^9^ and 10^10^ CFU/mL) treatment in Sprague-Dawley (SD) rats two weeks prior to challenge with *Salmonella*. The results showed that LAB can effectively inhibit the inflammatory index of TNF-α and IL-1β in rats [26].

Gastrointestinal epithelial cells provide a natural barrier against pathogen invasion when bacteria enter the intestine to avoid intestinal peristalsis excretion. The bacteria are then adsorbed to epithelial cells, which can maintain the balance of intestinal flora and reduce invasion. Adsorption of pathogenic bacteria can also promote the host immune function, so the adsorption capacity can be used as a screening of beneficial probiotic conditions of the host [27]. According to Pedersen and Tannock, each cell adsorbed more than 15 bacterial cells; this LAB strain was considered to have adsorption capacity [28]. Bacterial adhesion to cultured cells can be used to assess in vitro models of bacterial adherence to the intestinal mucosa; however, this does not account for the potential for adhesion to the mucus layer that covers the intestinal epithelial cells.

In this study, the ability of three strains of LAB to adhere to primary cultured epithelial cells and intestinal mucus was tested in mouse cells in an in vitro model. These experiments showed a strong correlation with in vitro models, indicating that LAB adhesion can co-present on the surface of epithelial cells and mucus in the small intestine. However, among the major criteria for selecting probiotics, adhesion to the intestinal epithelium is considered to be the most important. Adhesion to epithelial cells and/or mucus seems to mediate the colonization of the gastrointestinal tract by LAB and may be a prerequisite for competitive exclusion of gut pathogens and immune regulation of the host [29].

Kim et al. reported that *L. plantarum* lipotechoic acid (LTA) can prevent pathogen adhesion and internalization, and inhibit pathogen-induced expression of proinflammatory cytokines such as IL-8 and TNF-α [30]. LTA is a major component of the Gram-positive cell wall, and the structural and immunomodulatory effects of LTA vary widely among species. LTA from *L. plantarum* has been shown to be effective against pathogens [31]. Liu et al. showed a significant increase in IL-1β and IL-6 levels in the serum of mice two hours after the intraperitoneal injection of two strains of *V. alginolyticus*, ATCC 17749T and E0666 [32]. The results showed that IL-6 And IL-1β are important inflammatory cytokines that may play an important role in the inflammation caused by *V. alginolyticus* infection. When the body is invaded by pathogens, the first reaction is caused by inflammation due to pre-inflammatory cytokines, such as IL-6. Ritchie et al. showed that the lower half of the small intestine was the site of major colonization by *V. parahaemolyticus* (~10^9^ CFU/g) measured from homogenates in the middle and distal intestine [20]. In addition, histological analysis showed that more extensive histological changes occurred in the lower third of the small intestine. Candela et al. also demonstrated that IL-8 secreted by HT-29, and stimulated by TNF-α, IL-1β, and LPS, was effectively inhibited by both 10^6^ and 10^8^ CFU/mL of *B. longum* Bar33 and *L. acidophilus* Bar13 [33].

## 5. Conclusions

In this study, the effects of LAB and *V. parahaemolyticus* on the viability of the Caco-2 cell line were tested using an MTT assay. The results showed that LAB did not cause any damage to Caco-2 cells after two or four hours of co-culture, yet a four-hour incubation with *V. parahaemolyticus* (particularly the BCRC 10806 strain) caused damage to Caco-2 cells. In bream fillets, the three strains of LAB or their supernatant inhibited the two strains of *V. parahaemolyticus* successfully at 4 °C or at room temperature after a four-hour incubation. ELISA was used to detect proinflammatory cytokines expressed by different cell lines treated with LAB and *V. parahaemolyticus*. The results showed that TNF-α, IL-1β, and IL-6 were significantly inhibited by treatment with LAB. The three LAB strains effectively inhibited the expression of IL-8 mRNA induced by *V. parahaemolyticus* in both Caco-2 and HT-29 cells. In terms of RAW 264.7, the expression of IL-6 was also effectively inhibited. Animal experiments showed that the most effective bacterial strain in the intestinal epithelial cells and mucus of mice was *L. plantarum* PM 222. Pathological sections of mice in the small intestine showed that *L. plantarum* PM 222 had the greatest degree of protection of the small intestine with the fewest number of apoptotic cells.

## Figures and Tables

**Table 1:** The survival rate after *Vibrio parahaemolyticus* BCRC 10806 and BCRC 12865 were added to lactic acid bacteria in Tilapia fillet that was cultured for one or four hours at 4 °C and room temperature.

Strains	LAB	Survival of *Vibrio parahaemolyticus* (Log CFU/mL)							
		4 °C				Room Temperature			
		1 h		4 h		1 h		4 h	
		Culture	Supernatant	Culture	Supernatant	Culture	Supernatant	Culture	Supernatant
10806	Control	3.69 ± 0.08 ^a^	3.74 ± 0.05 ^a^	<1	<1	5.20 ± 0.01 ^a^	5.21 ± 0.01 ^a^	5.70 ± 0.03 ^a^	5.82 ± 0.04 ^a^
	*Lactobacillus plantarum* PM 222	<1 ^b^	<1 ^b^	<1	<1	3.81 ± 0.03 ^b^	4.52 ± 0.04 ^b^	<1 ^b^	<1 ^b^
	*Lactobacillus plantarum* LP 735	<1 ^b^	<1 ^b^	<1	<1	2.62 ± 0.02 ^d^	3.54 ± 0.04 ^d^	<1 ^b^	<1 ^b^
	*Lactobacillus plantarum* LP 010	<1 ^b^	<1 ^b^	<1	<1	2.79 ± 0.04 ^c^	3.66 ± 0.05 ^c^	<1 ^b^	<1 ^b^
12865	Control	4.28 ± 0.09 ^a^	4.23 ± 0.01 ^a^	3.73 ± 0.10 ^a^	3.74 ± 0.04 ^a^	5.95 ± 0.02 ^a^	6.01 ± 0.03 ^a^	6.55 ± 0.05 ^a^	6.61 ± 0.06 ^a^
	*Lactobacillus plantarum* PM 222	<1 ^b^	<1 ^b^	<1 ^b^	<1 ^b^	4.84 ± 0.03 ^b^	5.00 ± 0.03 ^b^	<1 ^b^	<1 ^b^
	*Lactobacillus plantarum* LP 735	<1 ^b^	<1 ^b^	<1 ^b^	<1 ^b^	3.83 ± 0.03 ^c^	3.90 ± 0.04 ^c^	<1 ^b^	<1 ^b^
	*Lactobacillus plantarum* LP 010	<1 ^b^	<1 ^b^	<1 ^b^	<1 ^b^	2.74 ± 0.06 ^d^	3.22 ± 0.11 ^d^	<1 ^b^	<1 ^b^

Bacteria counts were converted to log CFU/mL. Each value is expressed as mean ± standard deviation. ^a,b,c,d^ Values in the same column with different superscripts mean significant difference (*p* < 0.05).

**Table 2:** The survival rate after three lactic acid bacteria were added to *V. parahaemolyticus* BCRC 10806 and BCRC 12865 in Tilapia fillet that was cultured for one or four hours at 4 °C and room temperature.

Strains	Survival of *Lactic Acid Bacteria* (Log CFU/mL)							
	BCRC 10806				BCRC 12865			
	4 °C		Room Temperature		4 °C		Room Temperature	
	1 h	4 h	1 h	4 h	1 h	4 h	1 h	4 h
*Lactobacillus plantarum* PM 222	6.16 ± 0.02 ^Ab^	6.11 ± 0.01 ^Ac^	6.10 ± 0.02 ^Ac^	6.02 ± 0.02 ^Ac^	6.14 ± 0.04 ^Ab^	6.00 ± 0.01 ^Bc^	6.06 ± 0.06 ^Ab^	6.07 ± 0.00 ^Ac^
*Lactobacillus plantarum* LP 735	6.38 ± 0.01 ^Aa^	6.37 ± 0.01 ^Ab^	6.35 ± 0.02 ^Ab^	6.46 ± 0.09 ^Ab^	6.43 ± 0.00 ^Aa^	6.33 ± 0.01 ^Bb^	6.37 ± 0.02 ^Aa^	6.38 ± 0.02 ^Ab^
*Lactobacillus plantarum* LP 010	6.43 ± 0.02 ^Aa^	6.46 ± 0.00 ^Aa^	6.52 ± 0.00 ^Aa^	6.71 ± 0.03 ^Ba^	6.45 ± 0.02 ^Aa^	6.41 ± 0.01 ^Aa^	6.46 ± 0.02 ^Aa^	6.72 ± 0.01 ^Ba^

Bacteria counts are converted to log CFU/mL. Each value is expressed as mean ± standard deviation. ^A,B^ Values in the same row with different superscripts mean significant difference (*p* < 0.05). ^a,b,c^ Values in the same column with different superscripts mean significant difference (*p* < 0.05).

**Table 3:** Adhesive ability of *Lactobacillus* strains to mouse epithelial cells and mucus.

Species	Strain	Adhesive Ability	Intestinal Mucus (OD570 nm) ^§^
		Epithelial Cell (Bacteria per Cell) ^‡^	
*Lactobacillus plantarum*	PM 222	26.4 ± 9.6 ^a^	0.12 ± 0.03 ^a^
*Lactobacillus plantarum*	LP 010	18.7 ± 9.0 ^ab^	0.09 ± 0.01 ^b^
*Lactobacillus plantarum*	LP 735	14 ± 9.0 ^c^	0.05 ± 0.00 ^c^

Values with no common superscript letters differ significantly (*p* < 0.05). ^‡^ Adhesiveness of each strain is expressed as the mean number of attached bacteria in 10 randomly selected fields. ^§^ Standard deviation of OD570 nm readings from four replicate wells. ^a,b,c^ Values in the same column with different superscripts mean significant difference (*p* < 0.05).
